# Development of machine learning predictive models for estimating pharmaceutical solubility in supercritical CO_2_: case study on lornoxicam solubility

**DOI:** 10.3389/fchem.2025.1683695

**Published:** 2025-11-18

**Authors:** Luomeng Chao, Yongqiang Wang, Bayi Erta, Wei Guo, Haifeng Wang, Chelegeri Zhao, Yuxia Yang

**Affiliations:** 1 College of Animal Science and Technology, Inner Mongolia Minzu University, Tongliao, China; 2 Inner Mongolia Key Laboratory of Toxicant Monitoring and Toxicology, Tongliao, China; 3 Inner Mongolia Rambo Testing Technology Limited Company, Tongliao, China; 4 Zhalantun Vocational College, Zhalantun, China; 5 Tongliao Animal Disease Control Centof, Tongliao, China; 6 Tongliao Animal Quarantine Technology Service Center, Tongliao, China; 7 Tongliao Institute of Agricultural and Animal Husbandry Science, Tongliao, China; 8 Tongliao Animal Agriculture Development Service Center, Tongliao, China; 9 College of Computer Science and Technology, Inner Mongolia Minzu University, Tongliao, China

**Keywords:** machine learning, pharmaceuticals, prediction, nanotechnology, bioavailability

## Abstract

Production of nano-sized solid-dosage drugs is useful for pharmaceutical industry owing to high solubility and efficacy of the drugs for patients, which can also reduce the drugs side effects. For the solid-dosage oral formulations, the nanomedicine can be prepared via either top-down or bottom-up approach to enhance the drug solubility which in turns enhances the drug bioavailability. A novel methodology for simulation and prediction of medicine solubility in supercritical solvent was developed based on supervised learning algorithms for classification of the data. The data for the simulations were collected on solubility of a model drug in supercritical carbon dioxide. The supercritical-based processing is usually used for preparation of nanomedicine with enhanced bioavailability, and the developed simulation method can help design and optimize the process for industrial applications. The data was obtained with temperature and pressure as the input parameters, whereas the drug solubility is considered as sole estimated output in the model. The validation outputs indicated that great agreement was obtained between the measured data and the simulated values with acceptable regression coefficient for the whole simulations. The simulation results revealed that the supervised learning algorithm is robust and rigorous for prediction of drug solubility data in supercritical conditions and can be used for process optimization and understanding the effects of process parameters. This study is innovative as it methodically assesses diverse machine learning methodologies, encompassing polynomial regression at different complexity tiers and the Gaussian Process Regressor for predicting pharmaceutical solubility. This comparative framework illustrates the bias-variance tradeoff and offers pragmatic guidance for choosing suitable models according to dataset attributes. The methodology presents a time-efficient and cost-effective alternative to conventional thermodynamic modelling for supercritical pharmaceutical processing.

## Highlights

- Development of a method for preparation of nanomedicine.
- Artificial intelligence computational study on drug nanonization solubility.
- Validation and training the neural model using the measured data.

## 1 Introduction

Development of processes for enhancing drug solubility has been on the focus of research from pharmaceutical industry point of view. The majority of produced drug substances possess poor solubility in the body; thus, more dosage is taken to fulfil therapeutic effects (Lesutan et al., 2025). Therefore, the drug substances can be produced in manufacturing processes with ability to enhance the drug solubility. The preparation method for manufacturing of solid-dosage oral formulations is mainly based on solid-state processing such as granulation, milling, etc. Which are in batch mode of operations. Different techniques have been recently employed and tested for improvement of solubility of medicine in either batch or continuous mode such as cocrystal formation, nanocrystalline drugs, and salt formation (Huang et al., 2025; Sah et al., 2025; Serajuddin, 2007; Shaikh et al., 2019).

Among various processing techniques which have been developed for enhancing the drug solubility, nanodrug preparation has been more attractive due to superior properties of nano drugs such as easy delivery, high permeability, and high bioavailability. Indeed, the drugs can be prepared at nano scale by either top-down or bottom-up techniques. In the top-down approach, the synthesized drug at micron size is used to make it at nano or sub-micron size by different techniques such as milling (Carling and Brülls, 2021; Singh et al., 2018.; Li et al., 2015; Shaikh et al., 2021; Slámová et al., 2021). However, the process is based on solid-state processing, and it is difficult to control the process to achieve the desired products with enhanced properties. Therefore, the preparation method based on wet chemistry is preferred for production of drugs at nano size for enhanced pharmaceutical solubility.

Recently, scientists have developed pharmaceutical nanodrug processing based on supercritical technology in which the solvent is a supercritical gas, usually CO_2_ which is a good and safe solvent for pharmaceutical processing. The advantage of this supercritical processing is that no organic solvent is utilized which reveals that the process is green technology for preparation of nanomedicine at different scales (Obaidullah, 2023a; Obaidullah, 2023b; Sheikhi et al., 2025; Sofia et al., 2025). The process is considered to operate based on dissolution of the medicine in the solvent, and finally separation of the solvent which results in precipitation of nanoparticles of drug at the desired size. In order to control the size of drug particles, process understanding, and modeling is required to obtain the drugs at the desired size range (Liu et al., 2022).

The process modeling can be conducted in order to optimize the supercritical processing for preparation of nanomedicines. The models can be developed at different scales including mechanistic, thermodynamic, molecular, and machine learning. The most common method for simulation of supercritical-based processing of pharmaceuticals is based on thermodynamic approach in which equation of state (EoS) or activity coefficient methods or empirical correlations are developed and fitted to the measured data of solubility. The main aim of these models is to predict the drug solubility at various ranges of temperature and pressure, as the solubility is the most important factor in supercritical drug processing (He et al., 2025; Zhang et al., 2025).

Recently, Machine Learning (ML) based models have been successfully utilized for simulating chemical and biochemical processing applicable for various needs. These models can be employed for simulation of different systems such as fluid flow, solubility, and separation purposes (Alanaz et al., 2023; Yu et al., 2024). In this approach of modeling, observed data are employed for training the algorithm, and the trained model can be used for simulation of the process and finding the relationship between the model’s outputs and the underlying parameters. Therefore, these models can be utilized for process prediction as well as optimization. The main advantages of these modes are that they have great fitting capability for complex systems, and these models are not computationally expensive compared to mechanistic models such as CFD models and quantum chemical calculations.

Machine learning methods have demonstrated significant potential in predicting the solubility of pharmaceuticals under supercritical conditions. Advanced AI modeling has been effectively utilized to ascertain pharmaceutical solubility in supercritical processing for the manufacture of nanosized drug particles, exhibiting remarkable accuracy and efficiency (Obaidullah, 2023a). The development and validation of machine learning models for nanomedicine solubility in supercritical fluids have demonstrated efficacy in advanced pharmaceutical manufacturing (Liu et al., 2022). Extensive research demonstrates that the integration of diverse thermodynamic and hybrid machine learning methodologies yields accurate predictions of pharmaceutical solubility in supercritical fluids (Yu et al., 2024).

Researchers have developed novel machine learning methodologies to identify optimal values for critical parameters that enhance medication solubility in green chemistry solvents (Alanaz et al., 2023). Additionally, SVM-based machine learning models have been explicitly developed to assess the solubility of lornoxicam in supercritical solvents, illustrating the efficacy of supervised learning techniques for this pharmaceutical compound (Zhang and Mahdi, 2023). This research demonstrates that machine learning is an effective method for understanding and predicting the behavior of drugs under supercritical conditions. This is an exemplary approach to optimize pharmaceutical production operations.

The focus of this research is to develop a machine learning-based algorithm for prediction and simulation of medicine solubility in supercritical solvent at various temperature and pressure. The model is built to make the solubility of the drug in supercritical CO_2_ as a function of temperature and pressure. The model is based on support vector machine (SVM) for simulation of the solubility data. The drug model is Lornoxicam, and its solubility was predicted in the wide range of temperature and pressure using the developed SVM technique. Lornoxicam is a nonsteroidal anti-inflammatory (NSAID) derivative which is mainly taken for joint disorders. Increasing its solubility would cause lower dosage to be taken, and consequently less side effects (Pelalak et al., 2021).

## 2 Methods

**FIGURE 1:**
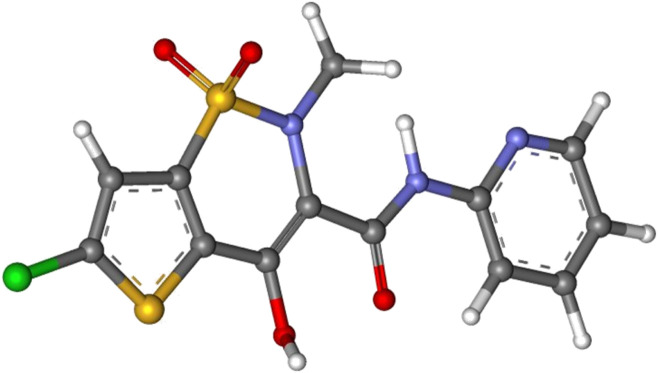
Lornoxicam structure used in this work.

The simulation in this work was carried out on a model drug, namely Lornoxicam which was processed in supercritical operation. Detailed explanation of the data acquisition and experiments are reported elsewhere (Pelalak et al., 2021). We have used the data here for simulation of the system and correlation of solubility data. The inputs to the developed model are temperature and pressure whose values are beyond the supercritical point of CO_2_. It is pointed out that the chemical structure of the medicine studied in this research is illustrated in Figure 1, and the drug chemical formula is C_13_H_10_ClN_3_O_4_S_2_.

## 3 Simulation methodology

The simulations were performed to make a relation between the process inputs (X) and the output (Y). The X parameters are indeed temperature and pressure, while the Y parameter is the medicine solubility in mole fraction unit (dimensionless). For fitting of the drug solubility data, support vector machine technique was used which is based on the supervised learning algorithms for prediction of process data. The main goal of the SVM method is to identify a hyperplane that, to the greatest degree feasible, partitions information elements into two groups, one group being of type one and the other unit being of type two. For potential matters, the method looks for a hyperplane that divides the set of linearly separable data, but for many real-world applications, the program seeks to maximize the soft margin to make the most accurate distinctions.

Support vector machines are often used for classification and regression, and they obtain very maximum performance on such tasks specifically for complex systems. The combination of several binary classifiers yields a multi-class SVM. For nonlinear issues, kernels improve SVMs, making them more versatile and able to tackle many types of scenarios. A decision surface just requires support vectors from the training data to be built. The remainder of the training data is therefore unimportant since once trained; the resulting model is perfect for code creation.

We employed SVM utilizing a Radial Basis Function (RBF) kernel for the dataset simulation. Kernels enhance the adaptability and functionality of SVMs, enabling their application across a wider range of contexts and situations. The Kernel Function is tasked with transforming polynomial order (Zhang and Mahdi, 2023).

## 4 Results and discussions

### 4.1 Experimental data analysis

**FIGURE 2:**
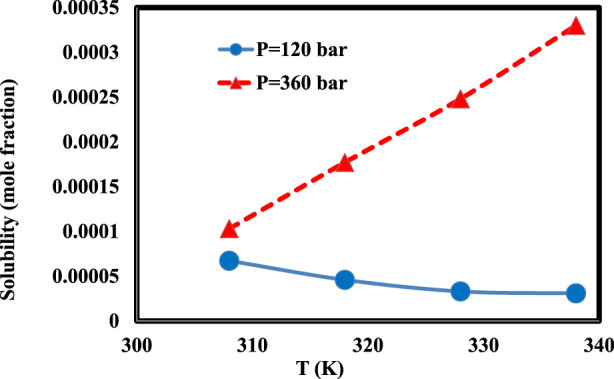
Solubility of drug *versus* the temperature and pressure.

In the first section, we analyzed the measured data of lornoxicam solubility collected from the source (Pelalak et al., 2021) to understand the effects of various parameters on the output parameter, Y. The results are indicated in Figure 2 for the solubility *versus* pressure and temperature. The data points are 32 at different conditions for lornoxicam between 308 and 338 K, at two distinct pressures of 120 and 360 bars (Pelalak et al., 2021). As observed in Figure 2, the solubility of lornoxicam has increased considerably when the pressure rises from 120 bar to 360 bar due to the major effect of pressure on medicine solubility. That can be said that the solubility trend changes when the pressure rises, due to the effect of pressure on the solubility (Liu et al., 2022). Indeed, at low pressure of 120 bar the solubility of lornoxicam is reduced with enhancing the *T* values, and the solubility is increased with temperature at high pressure of 360 bar (Pishnamazi et al., 2020). This behavior is due to the location of the cross-over pressure in the dataset which would change trend of solubility in the process (Pelalak et al., 2021).

### 4.2 Simulation results

**FIGURE 3:**
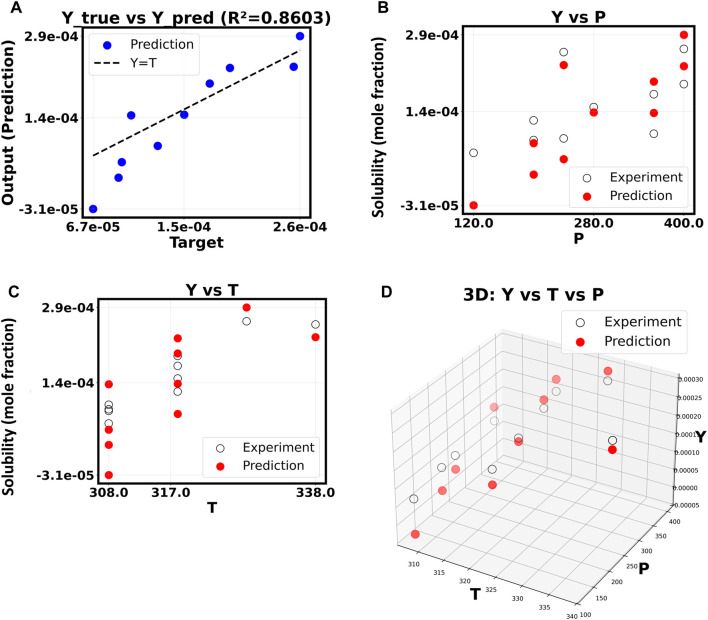
**(A–D)** Correlation of drug solubility (Y) based on P and T with SVM: polynomial order 1.

We used a first-order polynomial regression model to set performance standards in correlation of drug dataset. This linear model shows how Y changes when pressure (P) and temperature (T) change within the dataset. Figure 3 shows that this model does a better performance of predicting data than expected. The “Y true vs. Y pred” graph shows a strong linear relationship (*R* ^2^ = 0.8603) between the model’s predictions and the experimental goal values. The linear model can thus account for 86% of the variation in output. The “Y vs. P” and “Y vs. T” subplots are very important because they show that the red dots, which are the predictions, are very close to the overall trend of the experimental data. This means that the model has learned the basic, linear-like connections between Y and temperature and pressure.

**FIGURE 4:**
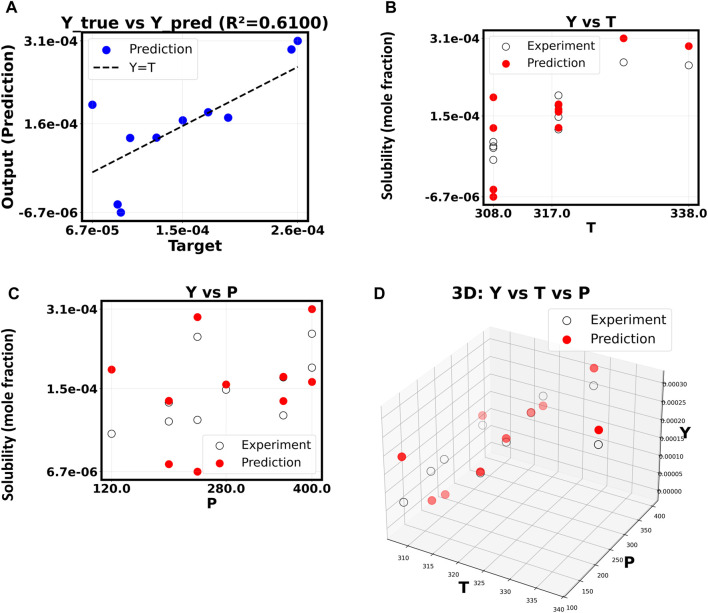
**(A–D)** Correlation of Y based on X. The polynomial order is 5.

**FIGURE 5:**
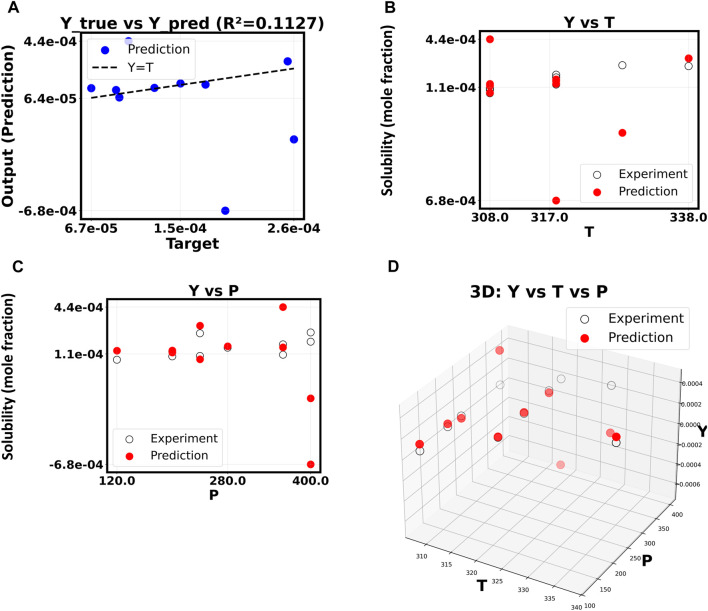
**(A–D)** Correlation of Y based on X. The polynomial order is 10.

The inquiry built on this good starting point to see if a more complex, non-linear model could find more subtle data relationships and improve the fitting results. By raising the polynomial degree to 5, the model can fit a more complex surface to the input feature space. Things become more complicated and useless as shown in Figure 4. Since *R* ^2^ dropped precipitously to 0.6100, the model had difficulty with the new test data. Overfitting, in which the machine learns false positives from the training data instead of the real signal, is the likely cause of this unexpected result. As the polynomial order increases beyond 10, this pattern becomes increasingly problematic (Figure 5). Now that the *R* ^2^ value has dropped to a very low 0.1127, the model is no longer able to generate accurate predictions of drug solubility. The “Y vs. P” and “Y vs. T” plots for this model show that the predictions do not match up with the experimental data at all, and they look random and scattered.

**FIGURE 6:**
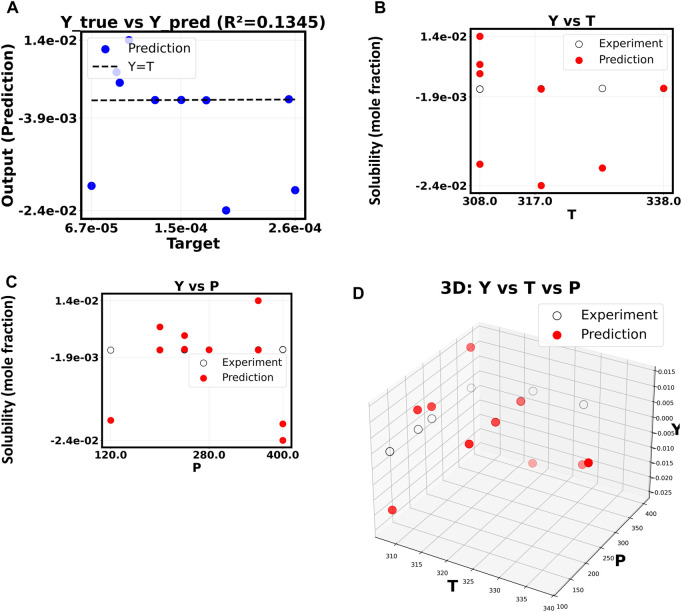
**(A–D)** Correlation of Y based on X. The polynomial order = 20.

**FIGURE 7:**
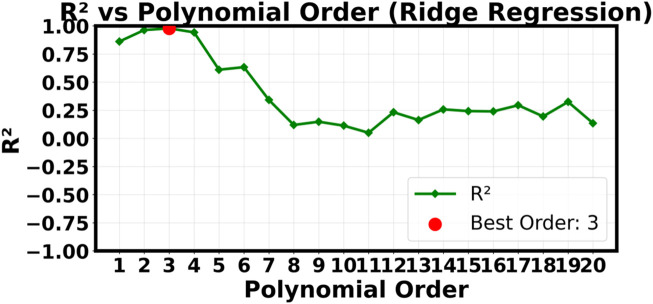
$R^{2}$ as a function of polynomial order.

**FIGURE 8:**
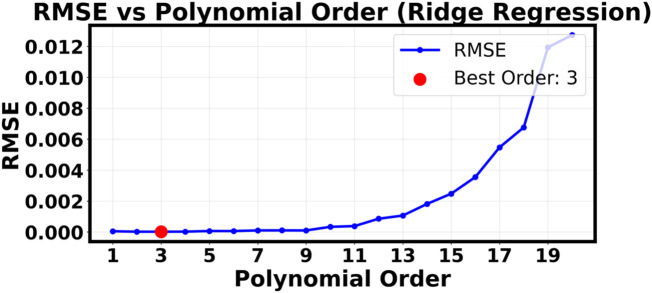
RMSE as a function of polynomial order.

**FIGURE 9:**
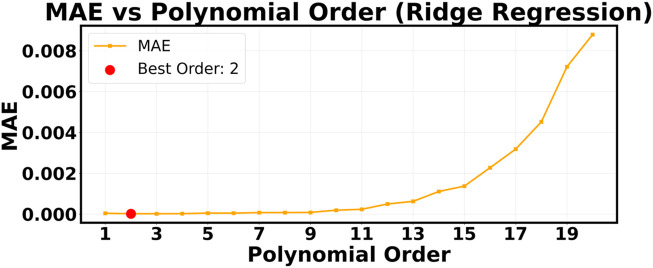
MAE as a function of polynomial order.

Figure 6, which shows 20th-order polynomial model, exemplifies the extreme case of overfitting. The model has completely failed to generalize in this case, as shown by the *R* ^2^ value of only 0.1345. The “Y true vs. Y pred” plot shows that the forecasts are all very close to each other in a narrow horizontal band. This means that the model is giving almost the same output value no matter what the input P and T values are. Figures 3, 6 show the successful linear model and the completely failed model, respectively. These figures provide strong visual evidence of the bias-variance tradeoff. The results confirm what we thought after our first hyperparameter optimization study (Figures 7–9), which showed that a low polynomial degree of 3 was the best choice for both peak performance (*R* ^2^ ≈ 1.0) and minimum error (RMSE/MAE). This shows that making the model too complicated is not an option and that a simpler, carefully chosen polynomial model is better for making reliable and accurate predictions about how the system will behave.

A Gaussian Process Regressor (GPR) was utilized to assess a non-parametric modeling approach. Polynomial models frequently encounter challenges when dealing with intricate data, while Gaussian Process Regressions (GPRs) demonstrate a remarkable ability to navigate these complexities effectively. The model utilized a composite kernel that combines Radial Basis Functions, Constant Kernels, and White Kernels, successfully tackling issues associated with noise, scaling, and signal covariance. A cross-validated grid search refined the essential hyperparameters of this kernel, such as the RBF length-scale and noise level, which contributed to the model’s robustness and its ability to generalize effectively.

**FIGURE 10:**
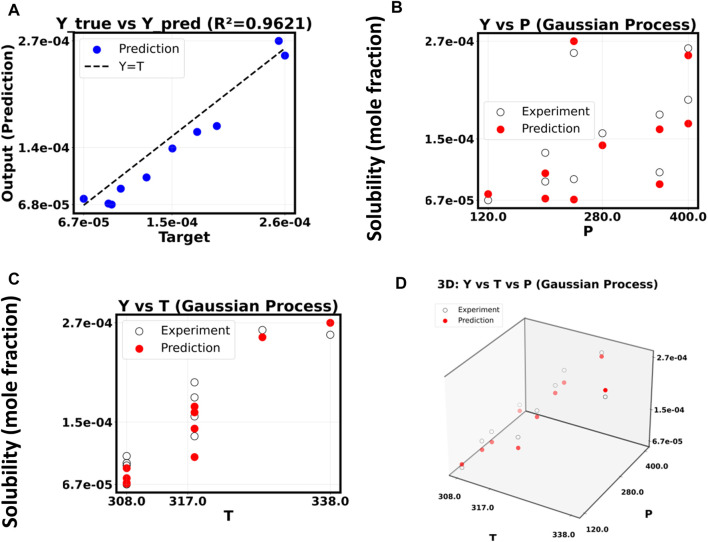
**(A–D)** Gaussian process regressor model.

Figure 10 shows that the GPR model performs exceptionally well. The polynomial models are sensitive, but the GPR model can explain almost 96% of the differences in the experimental results, which makes it more stable. The “Y true *versus* Y pred” subplot shows that the prediction points are very close to the ideal parity line (Y = T). This means that the prediction error is very low for all output values.

A more detailed examination of the model’s response to pressure (P) and temperature (T) substantiates its assertion of superiority. The “Y vs. P” and “Y vs. T” subplots show that the experimental data (black circles) and the GPR predictions (red dots) are very close to each other at every point. This means that the model has learned how to connect input situations to solubility of drug and general trends in a way that is not straightforward. The 3D graphic shows the expected and experimental markers close together in the P-T-Y space. This proves that the model can accurately fill in the gaps between data points. The Gaussian Process Regressor is a better and more reliable way to predict what will happen in this system because it does not fit high-order polynomial models too closely.

The developed models have the potential to enhance the processing conditions of supercritical CO_2_ in the realm of pharmaceutical manufacturing. This advancement aims to reduce the number of experimental trials required to identify the most effective operating parameters. The methodology has the potential for further enhancement by incorporating supplementary input variables, including co-solvent concentration, flow rate, and various chemical descriptors. Nonetheless, this expansion would increase the dimensionality of the feature space, thereby requiring more extensive experimental datasets to maintain the integrity of predictive accuracy. Our results indicate that increased model complexity must be judiciously matched with the available data to prevent overfitting. The comparative machine learning architecture developed herein offers a systematic methodology applicable to additional pharmaceutical substances and supercritical processing systems beyond Lornoxicam.

It is important to note that this study intentionally presents models across a wide range of complexity levels to demonstrate the bias-variance tradeoff in machine learning applications. While higher polynomial orders show declining performance due to overfitting, the optimized models demonstrate excellent predictive capability.

The first-order polynomial demonstrates robust baseline performance, signifying a strong linear relationship, whereas hyperparameter optimization reveals that modest polynomial complexity is optimal, resulting in nearly flawless prediction accuracy. The Gaussian Process Regressor delivers the most reliable and consistent forecasts, adeptly modeling the nonlinear solubility behavior while avoiding overfitting. These findings confirm that appropriate model selection, rather than just increasing complexity, is crucial for achieving reliable pharmaceutical solubility predictions under supercritical conditions.

## 5 Conclusion

This study successfully developed machine learning models to predict the solubility of Lornoxicam in supercritical CO_2_, employing temperature and pressure as input variables. The thorough assessment of various techniques, including polynomial regression models of varying complexities and the Gaussian Process Regressor, underscored the importance of suitable model selection. The first-order polynomial model demonstrated robust baseline performance, effectively capturing the essential linear connections in the solubility data. However, increasing polynomial complexity above optimal thresholds led to substantial overfitting, resulting in a notable decline in performance at higher polynomial degrees. This exemplifies the fundamental bias-variance tradeoff in machine learning applications. The Gaussian Process Regressor demonstrated superior performance, effectively modeling the complex nonlinear connection between process parameters and drug solubility across the experimental temperature and pressure ranges. Hyperparameter optimization indicated that modest polynomial complexity yields optimal performance. The generated models accurately forecasted the solubility behavior of Lornoxicam, encompassing the crossover pressure phenomenon where solubility trends inversely correlate with temperature at varying pressures. The models enhance the efficiency of experimental processes, save time and money in supercritical pharmaceutical manufacturing. The improved protocol for Lornoxicam, an NSAID employed in managing joint disorders, may enable reduced dosages and lessen side effects.

### 5.1 Future perspectives

The methodologies established in this study can be extended in various areas. Initially, employing the machine learning framework for supplemental pharmacological agents, particularly other NSAIDs and poorly soluble medications, would demonstrate its generalizability. Secondly, incorporating additional input variables such as co-solvent concentration, chemical descriptors, and structural features is expected to enhance predictive accuracy; nevertheless, larger datasets will be required to maintain model precision in higher-dimensional scenarios. Third, the amalgamation of these models with process optimization techniques should facilitate the systematic design of operational parameters for industrial-scale nanomedicine manufacturing. The integration of machine learning predictions with focused trials via active learning methodologies may alleviate experimental load while optimizing information acquisition. An expansion to anticipate supplementary process outcomes, such as particle size distribution and shape, would yield a comprehensive understanding of pharmaceutical manufacture with supercritical CO_2_ technology.

## Data Availability

The original contributions presented in the study are included in the article/supplementary material, further inquiries can be directed to the corresponding author.
